# A leukocyte activation test identifies food items which induce release of DNA by innate immune peripheral blood leucocytes

**DOI:** 10.1186/s12986-018-0260-4

**Published:** 2018-04-11

**Authors:** Irma Garcia-Martinez, Theresa R. Weiss, Muhammad N. Yousaf, Ather Ali, Wajahat Z. Mehal

**Affiliations:** 1 Department of Internal Medicine, Yale University School of Medicine, New Haven, CT USA; 2 Department of Pediatrics, Yale University School of Medicine, New Haven, CT USA

**Keywords:** Food sensitivity, Leukocyte activation test, Immune system

## Abstract

**Background:**

Leukocyte activation (LA) testing identifies food items that induce a patient specific cellular response in the immune system, and has recently been shown in a randomized double blinded prospective study to reduce symptoms in patients with irritable bowel syndrome (IBS). We hypothesized that test reactivity to particular food items, and the systemic immune response initiated by these food items, is due to the release of cellular DNA from blood immune cells.

**Methods:**

We tested this by quantifying total DNA concentration in the cellular supernatant of immune cells exposed to positive and negative foods from 20 healthy volunteers. To establish if the DNA release by positive samples is a specific phenomenon, we quantified myeloperoxidase (MPO) in cellular supernatants. We further assessed if a particular immune cell population (neutrophils, eosinophils, and basophils) was activated by the positive food items by flow cytometry analysis. To identify the signaling pathways that are required for DNA release we tested if specific inhibitors of key signaling pathways could block DNA release.

**Results:**

Foods with a positive LA test result gave a higher supernatant DNA content when compared to foods with a negative result. This was specific as MPO levels were not increased by foods with a positive LA test. Protein kinase C (PKC) inhibitors resulted in inhibition of positive food stimulated DNA release. Positive foods resulted in CD63 levels greater than negative foods in eosinophils in 76.5% of tests.

**Conclusion:**

LA test identifies food items that result in release of DNA and activation of peripheral blood innate immune cells in a PKC dependent manner, suggesting that this LA test identifies food items that result in release of inflammatory markers and activation of innate immune cells. This may be the basis for the improvement in symptoms in IBS patients who followed an LA test guided diet.

**Electronic supplementary material:**

The online version of this article (10.1186/s12986-018-0260-4) contains supplementary material, which is available to authorized users.

## Background

There are a multitude of commercially available blood tests purporting to diagnose food allergy [1], though most remain unvalidated [2]. Among the more widely utilized food sensitivity assays are leukocyte activation (LA) tests used to guide dietary advice used in complementary and alternative medicine practices [3, 4].

A number of small studies and abstracts have suggested clinical utility of dietary change using LA test results [5, 6] as well as reproducibility of test results, but are difficult to assess due to the lack of rigorous published methods. Previous literature also notes a lack of reproducibility in LA results and recommend against dietary guidance using these tests [3, 7–11].

LA tests assess changes in blood leukocyte populations in response to incubation with a large panel of standardized food antigens and are analyzed using a quantitative algorithm that assesses changes in number, cell volume, and conductance [12] after exposure to individual foods. More commonly utilized conventional food allergy tests examine the humoral, or antibody response (typically IgE), but do not provide information of the response on immune cells [13]. In contrast to a IgE-mediated food allergy, the term food sensitivity or intolerance often refers to non-IgE mediated symptomatic responses to food [14]. Guidelines sponsored by the National Institute of Allergy and Infectious Diseases note that diagnosing non-IgE-mediated immunologic adverse reactions to food can be challenging, and many immune cells may be involved in the pathogenesis of non-IgE-mediated food reactions [15].

A food sensitivity assay using only biological samples has a number of desirable attributes at a scientific and clinical level. Firstly, the results can be considered objective as the entire test is performed and reported without incorporating information about patient demographics, dietary profile, or symptom complex. This is an important point, as incorporation of such information can be used to reaffirm previously held concepts about associations between foods and symptoms, rather than providing new data that is based entirely on a biological test.

Our previous research assessing usage patterns of unconventional laboratory testing noted widespread use of LA tests Anecdotally, some clinicians report that elimination diets guided by LA test results were effective in treating chronic symptomatology, especially in conditions such as irritable bowel syndrome and dermatitis. Nevertheless, anecdotal reports and observational studies, despite encouraging results, cannot determine causality. Furthermore, retrospective studies are prone to bias [16]. Prospective randomized, controlled studies provide more rigorous data to determine the effects of an intervention. We recently completed a blinded, randomized controlled trial of IBS patients finding statistically significant improvements in patients with irritable bowel syndrome, compared to a sham diet, in response to dietary alterations guided by a LA test [17].

The objective of the current study was to bridge the gap in understanding between the known morphological and physiochemical (conductance) based changes which are detected by a commercially available LA test and current paradigms of immune activation. The immune system can be activated by a diverse range of stimuli and pathways. In recent years, there has been greater recognition of self-molecules that are typically sequestered inside cells inducing immune activation if they are extruded into the extracellular environment [18]. Release of internalized molecules can occur by non-specific cell death such as induced by physical trauma or thermal injury [19]. It can also occur by programmed release by immune cells to initiate an inflammatory response. Such molecules are generically termed damage associated molecular patterns (DAMPs), and currently over 20 such molecules have been identified.

We have identified the role of DNA as a DAMP in a number of disease states [18]. DNA is present inside the nucleus and mitochondria of healthy cells, and on extrusion can activate a specific receptor, Toll-like receptor 9 (TLR9) on immune cells [20]. This results in immune activation and the release of pro-inflammatory cytokines (IL-1β and TNF-α) and an anti-viral response (type-1 interferons). We have identified that release of DNA and activation of TLR9 is an important injury pathway in acetaminophen induced liver injury, pancreatitis and nonalcoholic steatohepatitis [21–23]. Others have shown this pathway to be important in systemic lupus erythematosus (SLE), and liver ischemia reperfusion injury [24, 25].

**Fig. 1:**
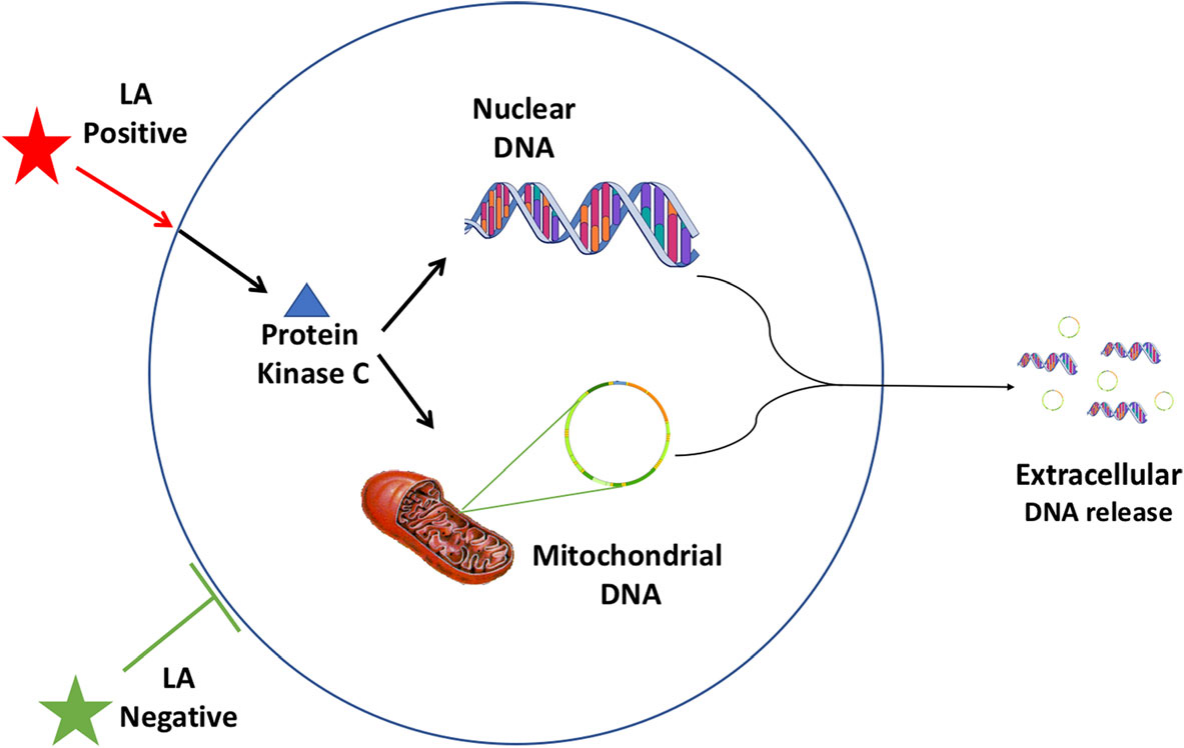
Proposed mechanism of immune pathway activated by food items that tested were identified by the leukocyte activation test as positive. The dependence on a protein kinase C pathway is a feature of DNA release by innate immune cells

**Fig. 2:**
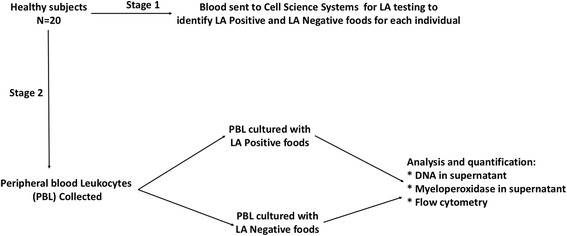
Experimental flow. Blood samples from 20 subjects were first sent for LA testing against a large panel of food items. After obtaining these results subsequent blood samples were incubated with LA positive and LA negative food items individually, and DNA release in the supernatant was assayed as explained in the methods. This was done in the absence can presence of signaling inhibitors

In this study, we assessed a commercially available LA test (Alcat Test, Cell Science Systems, Corp., Deerfield, FL). This test cultures peripheral blood leukocytes (PBL) with extracts from 200 individual foods, and identifies foods that induce changes in physiochemical properties in PBL. Based on changes in morphology and conductance, foods are categorized as *severe intolerance* (denoted as positive in this paper), moderate intolerance, mild intolerance, or acceptable foods/no reaction (denoted as negative in this paper). We hypothesized that positive foods result in release of DNA from peripheral blood leukocytes, resulting in an inflammatory response. Thus, positive food items are predicted to release DNA to a greater degree than negative food items (Fig. 1). We tested this hypothesis by initially obtaining LA test profiles of 20 healthy subjects by sending blood for Alcat testing. Subsequently, peripheral blood leukocytes, from the same subjects, were cultured with food items identified as positive or negative on LA testing, and DNA concentration in the cellular supernatant of immune cells quantified, in our independent academic laboratory. We further established whether this was a specific phenomenon and identified the major signaling pathways that were involved. (See experimental flow in Fig. 2).

## Methods

### Ethics statement

The study was approved by the Human Investigation Committee of Yale University (New Haven, CT). Blood and clinical data were collected at the Yale Center for Clinical Investigation outpatient facility. All subjects provided written informed consent.

### Participants

Eligible participants were healthy females (*n* = 17) and males (*n* = 3), with a mean age of 38 years of age (range 20 to 61 years) with no reported food allergies/sensitivities. Participants provided 5–11 blood samples (20 mL) approximately monthly for 17 months.

Exclusion criteria included a history of abdominal surgery (excluding cholecystectomy, appendectomy, hysterectomy, and hernia repair), inflammatory bowel disease, irritable bowel syndrome, and other major medical conditions (diabetes, hypertension, cardiovascular disease, and cancer). Other exclusion criteria were a history of radiation proctitis (or other known poorly controlled medical conditions that could interfere with bowel function). Antibiotic use within 1 month of enrollment, narcotic/opioid pain relievers, and other medications known to be affected by modest dietary change (such as warfarin and immunosuppressives such as cyclosporine) were criteria for exclusion. Participants were compensated $25 for each venipuncture.

All blood samples were collected at Yale University (New Haven, CT) and the initial sample (9 ml) was sent to the sponsor for LA testing, resulting in a list of foods categorized as positive, negative or intermediate for each subject. After obtaining this information, further blood samples were collected from the same subjects to conduct the experiments described below. All work apart from the initial LA testing was performed in the Section of Digestive Diseases at Yale University (New Haven, CT).

### Whole blood separation using Hetasep

Four tubes of blood preserved in sodium citrate for each participant were obtained and tested approximately 24 h after the blood was drawn. One milliliters of the separation medium Hetasep (HP, StemCell Technologies) was added to the 5 ml blood in the citrate tubes. The tubes were mixed gently and centrifuged at 90 g for 5 min at room temperature with the brake off. The samples were allowed to sit at room temperature for 10 min and the leukocyte-rich plasma layer was placed in a 50 ml tube and washed once with four-fold volume of saline (EKI Buffered Blood Bank Saline pH 7.0–7.2, item number: 12443-20 L). After centrifugation at 1200 rpm for 5 min the supernatant was transferred to a 50 ml tube and cell pellet re-suspended in 5 ml of saline. Cell count was checked and diluted with saline and HP.

### Incubation with positive or negative foods and inhibitors

Peripheral blood leukocytes (PBL) were cultured with positive or negative foods, replicating methods of the sponsor. Pre-prepared food extracts (the same panel of 200 foods as used in LA testing) were obtained from the sponsor (Additional file 1: Table S1). Standard commercially obtained food items were used for the test agent preparation. Briefly, food items were washed with water, cut into small pieces, and lyophilized. Upon completion of the freeze-drying process they were powdered, homogenized, and packaged. The resulting powder was used to prepare the primary stock solutions. For each test agent 2.5 g of powder was extracted using 50 ml of 50% ethanol solution for 1 h at room temperature with continuous agitation. Resulting extracts were vacuum filtered to remove the particulate matter using 0.45μn sterile filter assembly. From these solutions, 10× concentrated secondary stock solutions were prepared (concentrations used for the test were determined experimentally based on the original validations and/or verification performed throughout the product life cycle of the LA test) in 50% ethanol and filtered using 0.45μn sterile filter assembly. Working solutions were prepared as 10 times dilutions of the secondary stocks in the reagent grade water. They were applied to test cassettes which were then dried in a dehydration chamber for 18–24 h. at 100–105 °F and relative humidity of ≤20 to immobilize the test agents. Dried cassettes were capped and stored at room temperature until use.

All investigators were blinded to which food items were positive or negative for each subject’s PBL samples. PBL at 1 × 10^6^ cell/ml were plated and incubated with individual food items identified positive or negative for each participant for 45 min in EKI Buffered Blood Bank saline. One well was the negative control with PBL alone. Foods were diluted consistent with the methods of the sponsor (range between 1:4 and 1:1250). To identify any interactions between positive and negative foods in stimulating DNA release from PBL, co-cultures with these two types of foods was also performed. The following cell signaling inhibitors were added to the positive foods to see test if DNA release was dependent on these pathways: Ly294002 (InvivoGen, 50 μM), Bay11-7082 (InvivoGen, 10 μM), SP600125 (InvivoGen, 50 μM), SB202190 (InvivoGen, 20 μM), Go6983 (Tocris, 10 μM) and W7-Hydrocloride (Tocris, 50 μM).

Cells were incubated with a single positive (or negative) food with and without each inhibitor for 15 min at 37 °C in a shaker then for 30 min at room temperature. The supernatants were washed and stored at − 80 °C until their use.

### Quantification of supernatant total DNA

Quantification of plasma total DNA by direct fluorescent PicoGreen staining DNA quantification was performed using the PicoGreen dsDNA kit (Life Technologies), according to manufacturer instructions using 200 microl of supernatant. Each sample DNA was analyzed in two duplicated dilution series. Black microtiter plates were read in a plate reader (BioTek) at an emission wavelength of 520 nm and excitation of 480 nm.

### Quantification of supernatant myeloperoxidase (MPO)

The quantification of MPO in supernatants from cells treated with food extracts and inhibitors was performed by a standard ELISA using a commercial kit and 100 microl of supernatant (Human MPO ELISA Kit, Abcam).

### Flow cytometry

FASC data on PBL cell populations (50,000) was collected, and were gated based on the expression of the following surface markers:

- Neutrophils: CD66b+ CD16+
- Eosinophils: CD66b+ CD16 −
- Basophils: CD66b− CD16− CD123+

The CD63 activation marker (eBioscience) was analyzed in each population. Samples were acquired in a FacsCanto cytometer (Becton Dickinson) and the data were processed using the FacsDiva 12.0 software.

### Statistical analysis

Differences between the 2 groups were compared with Mann-Whitney U test. (GraphPad Prism 6; GraphPad Software Inc.,). An α value of 0.05 was the threshold for statistical significance. To compare the response of individual participant samples to positive and negative foods we determined which of the two food categories (positive or negative) gave greater DNA release in each participant. If test positivity or test negativity had no relevance to DNA release, the null scenario, we would expect an approximately even distribution, with each category of food giving greater DNA release approximately half the time. A statistically significant skewing from this would provide evidence of a selective response for either positive or negative foods.

## Results

The PBL leukocytes from the 20 subjects gave positive results to a range of food items as detailed in Additional files 2 and 3: Tables S2 and S3.

**Fig. 3:**
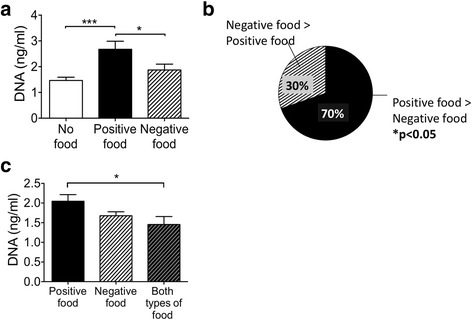
Positive foods result in greater release of DNA than negative foods. **a** Higher mean DNA concentration in supernatants of PBL cultured with positive as compared to negative food items. Peripheral blood was obtained, immune cells isolated and cultured with known positive or negative food items. Supernatant was collected and DNA concentration was assayed using PicoGreen staining. (*** *P* < 0.005, and * *P* < 0.05). **b** For individual blood samples positive foods resulted in a higher supernatant DNA concentration than negative foods 70% of the time (*P* < 0.05). **c** Exposure of cells to positive and negative foods resulted in a DNA release response that was similar to the negative food alone. (* *P* < 0.05). Nineteen patients were included in two different times and two positive and two negative foods were studied for each patient each time, resulting in the following sample sizes: “No Food Group” *n* = 78, “Positive food Group” *n* = 78, “Negative Food Group” *n* = 78. Data are represented as mean with SEM

There was a higher concentration of DNA in the supernatants of cells incubated with positive compared to negative foods, or no food (Fig. 3a). For 53 out of a total of 76 assays (70%), the positive foods gave a higher supernatant DNA content than the negative food in cells from the same participant (Fig. 3b). This result is statistically significant with *P* < 0.05. To determine which of the response to positive or negative foods was dominant we also performed a co-culture with one of each category (positive or negative) food. The results (Fig. 3c) show that co-culture of cells with positive and negative foods brings the response towards negative food (less DNA release).

**Fig. 4:**
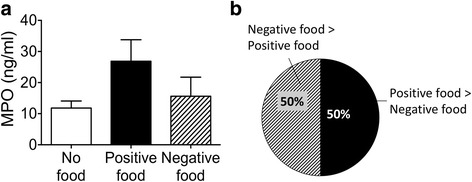
Positive foods do not result in greater release of myeloperoxidase than negative foods. **a** No difference in average myeloperoxidase (MPO) concentration in supernatants of blood immune cells cultured with positive or negative food items. Peripheral blood was obtained, immune cells isolated and cultured with known positive or negative food items. Supernatant was collected and MPO concentration was assayed using a commercial ELISA. **b** For individual blood samples positive and negative foods resulted in greater MPO concentrations an identical number of times. Nineteen patients were included in two different times and two positive and two negative foods were studied for each patient each time, resulting in the following sample sizes: “No Food Group” *n* = 78, “Positive food Group” *n* = 78, “Negative Food Group” *n* = 78. Data are represented as mean with SEM

To establish if this result of greater DNA release by positive samples is specific to DNA release, or if it is a general, and possibly non-specific phenomenon, we quantified myeloperoxidase (MPO) in cellular supernatants exposed to positive and negative foods. Figure 4a shows the average MPO concentration in supernatants exposed to both categories of foods, and Fig. 4b the numbers of tests in which the positive food had a higher MPO concentration than the negative, and vice versa. By both criteria, positive foods did not result in significantly greater release of MPO. These results demonstrate some specificity for DNA release by positive food items.

**Fig. 5:**
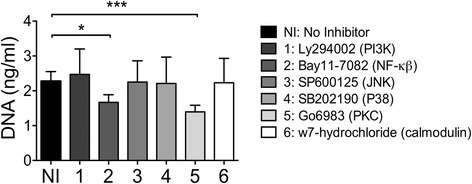
Protein kinase C and NF-kb inhibition reduce DNA release by positive foods. All samples were treated with a positive food with either no inhibitor, or one of the signaling inhibitors listed. Inhibitor #2 (Bay11-7082, inhibits NF-kb) and Inhibitor #5 (Go6983, inhibits PKC) resulted in resulted in a significant decrease in mean DNA concentration when added to ALCAT positive foods. (*** *P* < 0.005, and * *P* < 0.05). Ten patients were included and two positive foods alone or with inhibitors were tested. Data are represented as mean with SEM

To further characterize whether the release of DNA by immune cells in response to positive foods is a specific phenomenon, and to identify the signaling pathways that are required for DNA release, we tested if specific inhibitors of key signaling pathways could block DNA release. Inhibitors specific for Phosphoinositide 3-kinase (PI3Kinase; inhibitor Ly294002), nuclear factor-kβ (NF-kβ, Bay 11-7082), c-Jun N-terminal kinase (JNK; SP600125), mitogen-activated protein kinase P38 (MAPK P38; SB202190), protein kinase C (PKC; Go6983) and calmodulin (W7-hydrochloryde) were tested. As can be seen from Fig. 5, the PKC inhibitor Go6983 and the NF-kβ inhibitor Bay11-7082 resulted in significant reduction of positive food-stimulated DNA release [26, 27].

**Fig. 6:**
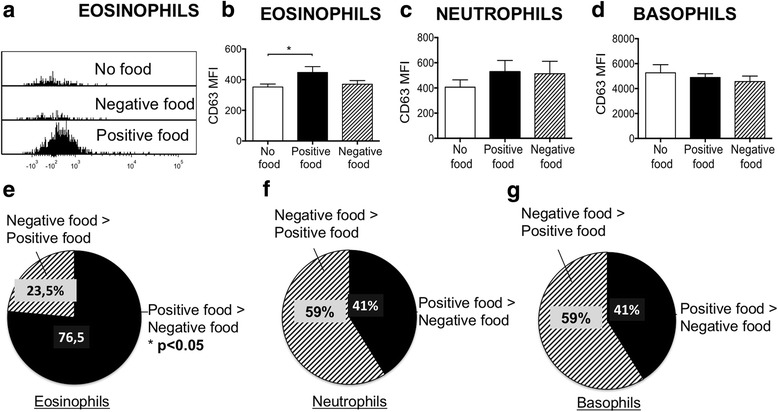
Positive foods result in upregulation of CD63 in eosinophils to a greater degree than negative foods. **a** Histogram of CD63 levels on an eosinophil population after exposure to no food, negative food and positive food, showing a shift to the right for the positive foods. **b**, **c** and **d** Histogram of meal fluorescence intensity (MFI) of CD63 for eosinophils, neutrophils and basophils. **e** Eosinophils from subjects gave a higher CD63 level with positive as compared to negative food items (* *P* < 0.05). **f** and **g** Neutrophils and basophils did not have a higher level of CD63 with positive foods. Ten patients were included and two positive and two negative foods were studied for each patient, resulting in the following sample sizes: “No Food Group” *n* = 18, “Positive food Group” *n* = 18, “Negative Food Group” *n* = 18. Data are represented as mean with SEM

Finally, we wished to identify whether a particular immune cell population was activated by positive food items. As the activation step occurs 45 min after adding food, it is highly unlikely that this phenomenon involves T cells or B cells (that take many hours and days to activate). We therefore focused on innate cells that can be activated in minutes. LA testing was performed in the standard manner, and neutrophils, eosinophils, and basophils were identified by established cell surface markers using flow-cytometric analysis, and up-regulation of CD63 was used as a marker of activation (Additional file 4: Figure S1). Positive samples resulted in a median CD63 levels greater than no food for eosinophils, but not for neutrophils or basophils (Fig. 6b-d). Data was also analyzed to quantify the number of times positive foods gave a CD63 level greater than negative foods. For individual subjects, positive food samples resulted in a greater CD63 level than negative food samples on eosinophils in 76,5% of tests (*p* < 0.05) (Fig. 6e), but only 41% of the time for neutrophils and basophils (not significant) (Fig. 6f and g).

## Discussion

The immune system identifies self (safe) from non-self (pathogen), and subsequently mounts a variety of responses against non-self. Nutrients occupy a unique place, because despite being foreign (non-self) substances they routinely enter the body through normal food intake. This poses a challenge for the immune system, and the reasons why the immune system usually does not mount an immune response are not fully understood. Food allergies (as defined by a positive IgE response to specific foods) do however occur, and respond to diets eliminating the food in question. An IgE response is however just one form of immune response, and non-IgE mediated immune responses (sometimes referred to as food intolerance) may be responsible for a wide range of clinical symptoms associated with the consumption of foods. One of the challenges in the field of non-IgE mediated food intolerance is the lack of standardized testing. LA testing has been clinically used for 30 years, and its utility has been examined in a number of small, uncontrolled, retrospective studies [6, 8, 9]. Recently we reported on a prospective, double blinded randomized study of a LA test in which subjects with IBS who followed LA test based dietary guidance had a significant improvement in the primary outcome of IBS Global Improvement Scale, as compared to participant who did not follow a LA test based dietary guidance [17]. This is the first rigorously designed and conducted high quality clinical study and demonstrates that the LA testing is dealing with a biological effect.

Our goal was to identify known immunological phenomenon that are independent of an IgE response and are identified by LA testing. DNA is typically intracellular, but can be released into the extracellular environment in a dysregulated way by cell death, and also in a regulated way such as the release of DNA neutrophil extracellular traps (NETS) by neutrophils, and many cells in micro-particles [28]. This extracellular DNA can have many functions, one of which is the activation of pattern recognition receptors such as TLR9, resulting in an inflammatory response. Collectively our data shows that food items that have been identified by LA testing as being positive for a particular subject result in greater DNA release into the extracellular environment from peripheral blood leukocytes than food items that have been identified as negative (Fig. 3). Furthermore, the release of DNA by positive foods appears to be a specific phenomenon as it does not extend to myeloperoxidase (Fig. 4), suggesting that that DNA release is not simply due to cell death. The fact that the DNA release is a specific process is further supported by the ability of a PKC and NF-kβ inhibitors to block DNA release by positive food items (Fig. 5). PKC activation is well known to regulate a wide range of immune cell functions ranging from activation, to polarity, to division [29]. Of relevance to this study PKC activation has been previously shown to be required for the release of DNA Nets by neutrophils, and our study now provides supporting data for a role of PKC in DNA release by eosinophils [30]. The ability of both a protein Kinase C and an NF-kβ inhibitor to block DNA release may be due to both being on the same pathway, and PKC has previously been shown to phosphorylate and activate IκB kinase complex (IKK) resulting in translocation of NF-kβ to the nucleus [31].

The mechanism of eosinophil activation by foods and other stimuli is poorly understood. The current study was designed to identify pro-inflammatory pathways such as DNA release associated with LA testing, and the mechanism of eosinophil activation by food items is still an open question. This is true for even well characterized eosinophil conditions such as eosinophilic esophagitis [32]. It is however notable here that an elemental diet can result in improvement in eosinophilic esophagitis in children and adults, clearly suggesting a food driven response [33]. The identity of the components of each food that are responsible LA test responses is not yet known. Although significant degradation of food molecules occurs during passage along the GI tract many molecular structures remain intact, with gluten being the best known example. The food interaction relevant to IBS may be occurring in the gut associated lymphoid system which contains eosinophils. Release of DNA in the form of cells is best characterized for neutrophils through a process of cytolytic extracellular trap cell death (ETosis), and this has also been described for human eosinophils [34]. ETosis was detected from in vivo specimens, and could also be stimulated in vitro by a wide range of stimuli including calcium ionophores, phytohemagglutinin and immobilized IgG, and resulted in eosinophil death [34]. Eosinophils express several TLRs including TLR7 and TLR9. Stimulation of TLRs and other pattern recognition receptors induces a wide range of responses including oxidative burst, activation of the adhesion system and release of pro-inflammatory cytokines [35]. This opens up the possibility of the released DNA activating adjacent neutrophils, and amplifying the original stimulus. This could be tested by using TLR9 antagonists which are being developed for clinical use in sterile inflammation [36].

A number of diseases are thought to have DNA mediated activation of the immune system as an important component of their pathogenesis. These include systemic lupus erythematosus (SLE), liver ischemia reperfusion injury, acetaminophen induced liver injury, and non-alcoholic steatohepatitis [21, 23–25]. These are characterized by systemic inflammation and the presence of cell free DNA in the circulation. The demonstration that certain foods can also result in release of DNA from peripheral blood leukocytes may provide an important new signal for activation of a DNA mediated immune response.

Since cell free DNA is known to be associated with inflammation [36], one implication of these findings is that DNA release following consumption of specific food items may result in inflammation and associated symptoms. Since the highest concentration of food constituents is in the gastrointestinal tract and liver, the majority of food stimulated DNA release, inflammation and symptoms may be in these organs. Among the known diseases which may be impacted by this process is eosinophilic esophagitis, which is a chronic immune disease of the esophagus and can result in dysphagia and strictures [37]. In support of the proposition that food stimulated DNA release by eosinophils may be driving esophageal inflammation is the fact that the esophagus is exposed to the highest concentration of food constituents, the increase in eosinophils seen on histology of esophageal mucosa, the production of inflammatory cytokines by esophageal epithelium on activation of TLRs, and the demonstration that removal of food items by placing participants on an elemental diet results in improvement of symptoms and histology [37–39]. Furthermore, conventional IgE-mediated allergy responses have been shown to be dependent on TLR9 [40]. Whether LA testing can identify individual food items which trigger eosinophilic esophagitis remains to be tested.

The development of NASH has strong dietary associations, and we have recently shown that plasma DNA is a driver of inflammation in NASH [23, 41]. Changes in diet have been shown to result in significant improvement in NASH histology, and it has been assumed that this improvement is likely attributable to weight loss induced by dietary changes. It is however possible that removal of specific food items may also have contributed to the improvement in NASH. Since long-term compliance with low calorie diets is very difficult, it would be of great interest to test if removal of specific foods which may be triggering release of DNA into the circulation can improve NASH.

## Conclusion

This is the first demonstration that specific foods can result in release of DNA by peripheral blood leucocytes, and further that these items can be identified by a peripheral blood LA test. This may provide a mechanistic rationale for the reported findings of clinical improvement in patients using LA tests to guide dietary choices, and provides support for conducting randomized clinical trials of these tests in gastrointestinal and liver diseases known to have dietary associations.

## Additional files

Additional file 1: **Table S1.** List of foods items used for standard Alcat testing. (DOCX 20 kb)

Additional file 2: **Table S2.** Occurrence of specific positive foods identified. (DOCX 16 kb)

Additional file 3: **Table S3.** Positive Foods Identified per Subject. (DOCX 21 kb)

Additional file 4: **Figure S1.** Representative cell gating and CD63 expression. (A) Gating of total cells, eosinophils, neutrophils and basophils for a representative sample. (B) Basophils, eosinophils and neutrophils CD63 expression for a representative sample no treated (no food) or treated with a positive food (red food). (TIFF 8103 kb)

## Abbreviations

DAMPs: Damage associated molecular patterns
JΝΚ: c-Jun N-terminal kinase
LA: Leukocyte activation
MAPK: Mitogen-activated protein kinase
MFI: Meal fluorescence intensity
MPO: Myeloperoxidase
NASH: Nonalcoholic steatohepatitis
NETS: Neutrophil extracellular traps
NF-kβ: Nuclear factor-kβ
PBL: Peripheral blood leukocytes
PI3 Kinase: Phosphoinositide 3-kinase
PKC: Protein kinase C
SLE: Systemic lupus erythematosus
TLR9: Toll-like receptor 9
