# Supplementary material for: A leukocyte activation test identifies food items which induce release of DNA by innate immune peripheral blood leucocytes
Source: Nutr Metab (Lond). 2018 Apr 11;15:26. doi: 10.1186/s12986-018-0260-4 (PMC5896029; doi:10.1186/s12986-018-0260-4)
Supplement: Supplementary file 1 — Table S1. List of foods items used for standard Alcat testing. (DOCX 20 kb) [file 12986_2018_260_MOESM1_ESM.docx]

| Apple | Celery | Honeydew Melon | Pork | Tapioca |
| --- | --- | --- | --- | --- |
| Almond | Cherry | Jalapeno Pepper | Papaya | Thyme |
| Asparagus | Clam | Kidney Bean | Paprika | Trout |
| Avocado | Coconut | Kiwi | Peppermint | Turnip |
| Amaranth | Codfish | Kale | Pistachio | Turmeric |
| Apricot | Coffee | Kelp | Pumpkin | Tilapia |
| Artichoke | Crab | Lamb | Parsnip | Vanilla |
| Acorn Squash | Cranberry | Iceberg Lettuce | Pine Nut | Veal |
| Anchovy | Cucumber | Lemon | Pomegranate | Venison |
| Barley | Caraway | Lime | Quinoa | Wheat |
| Beef | Cayenne Pepper | Lobster | Portobello Mushroom | White Potato |
| Broccoli | Chickpea | Lentil Bean | Rye | Watermelon |
| Baker's Yeast | Clove | Lima Bean | Radish | Whey |
| Banana | Cumin | Leaf Lettuce | Raspberry | Walnut |
| Black Pepper | Canola Oil | Leek | Red Beet/Beet Sugar | Wild Rice |
| Brown/White Rice | Cardamom | Licorice | Romaine Lettuce | Watercress |
| Butternut Squash | Catfish | Mustard | Rosemary |  |
| Basil | Chamomile | Millet | Shrimp |  |
| Bell Pepper Variety | Chicken Liver | Mushroom | Soybean |  |
| Blueberry | Chili Pepper | Malt | Sweet Potato |  |
| Brewer's Yeast | Coriander | Mango | Salmon |  |
| Brussel Sprouts | Date | Macadamia Nut | Scallop |  |
| Bay Leaf | Dill | Mackerel | Sesame |  |
| Black Beans | Duck | Mahi Mahi | Snapper |  |
| Blackberry | Egg White | Maple Sugar | Sole |  |
| Black-Eyed Pea | Egg Yolk | Mung Bean | Spinach |  |
| Buckwheat | Eggplant | Mussel | Saffron |  |
| Black Currant | Endive | Navy Bean | Scallions |  |
| Bok Choy | Fructose | Nutmeg | Sheep's Milk |  |
| Brazil Nut | Fig | Nectarine | Sorghum |  |
| Buffalo | Flaxseed | Oat | Spelt |  |
| Cabbage | Fava Bean | Onion | Squid |  |
| Cantaloupe | Fennel Seed | Orange | Swiss Chard |  |
| Casein | Flounder | Olive | Swordfish |  |
| Cane Sugar | Ginger | Oregano | Safflower |  |
| Carrot | Goat's Milk | Oyster | Sage |  |
| Corn | Grapefruit | Okra | Sardine/Herring |  |
| Cow's Milk | Garlic | Parsley | Sea Bass |  |
| Cauliflower | Gluten/Gliadin | Peach | Sunflower |  |
| Chicken | Grape | Pecan | Yellow Squash |  |
| Cinnamon | Green Pea | Pineapple | Strawberry |  |
| Cocoa | Halibut | Pinto bean | String Bean |  |
| Cottonseed | Honey | Plum | Tomato |  |
| Candida Albicans | Hops | Psyllium | Tuna |  |
| Carob | Haddock | Peanut | Turkey |  |
| Cashew | Hazelnut | Pear | Tea |  |

**Additional file 1: Table S1. List of foods items used for standard Alcat testing**
