# Supplementary material for: A leukocyte activation test identifies food items which induce release of DNA by innate immune peripheral blood leucocytes
Source: Nutr Metab (Lond). 2018 Apr 11;15:26. doi: 10.1186/s12986-018-0260-4 (PMC5896029; doi:10.1186/s12986-018-0260-4)
Supplement: Supplementary file 2 — Table S2. Occurrence of specific positive foods identified. (DOCX 16 kb) [file 12986_2018_260_MOESM2_ESM.docx]

**Additional file 2: Table S2. Occurrence of specific positive foods identified**

| **Severe (Positive) Foods Identified in Samples** | **Occurrence** | **Severe (Positive) Foods Identified in Samples** | **Occurrence** |
| --- | --- | --- | --- |
| Apple | 1 | Iceberg lettuce | 3 |
| Artichoke | 3 | Jalapeno pepper | 1 |
| Avocado | 1 | Kelp | 4 |
| Banana | 1 | Kiwi | 1 |
| Barley | 5 | Licorice | 1 |
| Basil | 2 | Lobster | 1 |
| Black pepper | 1 | Malt | 4 |
| Black/green tea | 1 | Mussel | 1 |
| Brewer’s yeast | 5 | Mustard seed | 2 |
| Brussels sprouts | 1 | Nutmeg | 1 |
| Buckwheat | 2 | Onion | 1 |
| Cabbage | 1 | Oregano | 1 |
| Canola oil | 2 | Papaya | 1 |
| Cantaloupe | 2 | Peach | 1 |
| Caraway | 2 | Peanut | 1 |
| Carob | 1 | Pear | 1 |
| Cashew | 1 | Pecan | 1 |
| Cauliflower | 5 | Peppermint | 2 |
| Cinnamon | 1 | Pinto bean | 1 |
| Clove | 1 | Plum | 1 |
| Cocoa | 1 | Pumpkin | 2 |
| Coconut | 1 | Rice | 1 |
| Codfish | 2 | Rosemary | 1 |
| Coffee | 3 | Rye | 4 |
| Coriander | 1 | Sage | 1 |
| Corn | 2 | Scallions | 2 |
| Cottonseed | 1 | Sesame | 5 |
| Cow’s milk | 2 | Soybean | 1 |
| Cucumber | 2 | Spelt | 4 |
| Cumin | 2 | Strawberry | 1 |
| Egg white | 1 | Thyme | 1 |
| Fructose (HFCS) | 1 | Tomato | 4 |
| Ginger | 1 | Turkey | 1 |
| Grape | 5 | Turmeric | 2 |
| Honey | 1 | Vanilla | 2 |
| Honeydew melon | 1 | Wheat | 4 |
| Hops | 4 | Zucchini squash | 1 |
