# Supplementary material for: A leukocyte activation test identifies food items which induce release of DNA by innate immune peripheral blood leucocytes
Source: Nutr Metab (Lond). 2018 Apr 11;15:26. doi: 10.1186/s12986-018-0260-4 (PMC5896029; doi:10.1186/s12986-018-0260-4)
Supplement: Supplementary file 3 — Table S3. Positive Foods Identified per Subject. (DOCX 21 kb) [file 12986_2018_260_MOESM3_ESM.docx]

**Additional file 3: Table S3. Positive Foods Identified per Subject**

| **Subjects** | **Positive Foods** | | | | | | | | | | | | | |
| --- | --- | --- | --- | --- | --- | --- | --- | --- | --- | --- | --- | --- | --- | --- |
|  | **Total** | **1** | **2** | **3** | **4** | **5** | **6** | **7** | **8** | **9** | **10** | **11** | **12** | **13** |
| **01** | 1 | Papaya |  |  |  |  |  |  |  |  |  |  |  |  |
| **02** | 9 | Artichoke | Brussels  Sprouts | Coffee | Coriander | Grape | Licorice | Pinto Beans | Scallions | Turkey |  |  |  |  |
| **03** | 3 | Avocado | Corn | Egg White |  |  |  |  |  |  |  |  |  |  |
| **04** | 10 | Barley | Basil | Brewer’s Yeast | Cauliflower | Grape | Honey | Peach | Pumpkin | Rice | Tomato |  |  |  |
| **05** | 4 | Cabbage | Cucumber | Grape | Pear |  |  |  |  |  |  |  |  |  |
| **06** | 5 | Artichoke | Canola Oil | Kelp | Malt | Tomato |  |  |  |  |  |  |  |  |
| **07** | 4 | Cauliflower | Codfish | Hops | Mussel |  |  |  |  |  |  |  |  |  |
| **08** | 9 | Banana | Black Pepper | Cauliflower | Codfish | Coffee | Corn | Ginger | Hops | Turmeric |  |  |  |  |
| **09** | 6 | Basil | Mustard Seed | Onion | Pumpkin | Scallions | Zucchini Squash |  |  |  |  |  |  |  |
| **10** | 7 | Canola Oil | Cauliflower | Cow’s Milk | Cumin | Rosemary | Tomato | Turmeric |  |  |  |  |  |  |
| **11** | 7 | Barley | Cow’s Milk | Cucumber | Lobster | Rye | Spelt | Wheat |  |  |  |  |  |  |
| **12** | 4 | Brewer’s Yeast | Coffee | Hops | Kelp |  |  |  |  |  |  |  |  |  |
| **13** | 13 | Apple | Artichoke | Buckwheat | Cantaloupe | Caraway | Cauliflower | Iceberg Lettuce | Kiwi | Nutmeg | Oregano | Sage | Thyme | Vanilla |
| **14** | 9 | Barley | Brewer’s Yeast | Kelp | Malt | Peppermint | Rye | Sesame | Spelt | Wheat |  |  |  |  |
| **15** | 11 | Barley | Black/Green Tea | Buckwheat | Caraway | Carob | Coconut | Cumin | Malt | Rye | Spelt | Wheat |  |  |
| **16** | 9 | Brewer’s Yeast | Cantaloupe | Grape | Honeydew Melon | Iceberg Lettuce | Kelp | Peanut | Plum | Vanilla |  |  |  |  |
| **17** | 6 | Cashew | Cinnamon | Jalapeno Pepper | Mustard Seed | Sesame | Tomato |  |  |  |  |  |  |  |
| **18** | 8 | Cocoa | Cottonseed | Fructose (HFCS) † | Grape | Hops | Sesame | Soybean | Strawberry |  |  |  |  |  |
| **19** | 3 | Brewer’s Yeast | Clove | Sesame |  |  |  |  |  |  |  |  |  |  |
| **20** | 9 | Barley | Iceberg Lettuce | Malt | Pecan | Peppermint | Rye | Sesame | Spelt | Wheat |  |  |  |  |

† High fructose corn syrup
