# Supplementary figures and images for: A leukocyte activation test identifies food items which induce release of DNA by innate immune peripheral blood leucocytes
Source: Nutr Metab (Lond). 2018 Apr 11;15:26. doi: 10.1186/s12986-018-0260-4 (PMC5896029; doi:10.1186/s12986-018-0260-4)

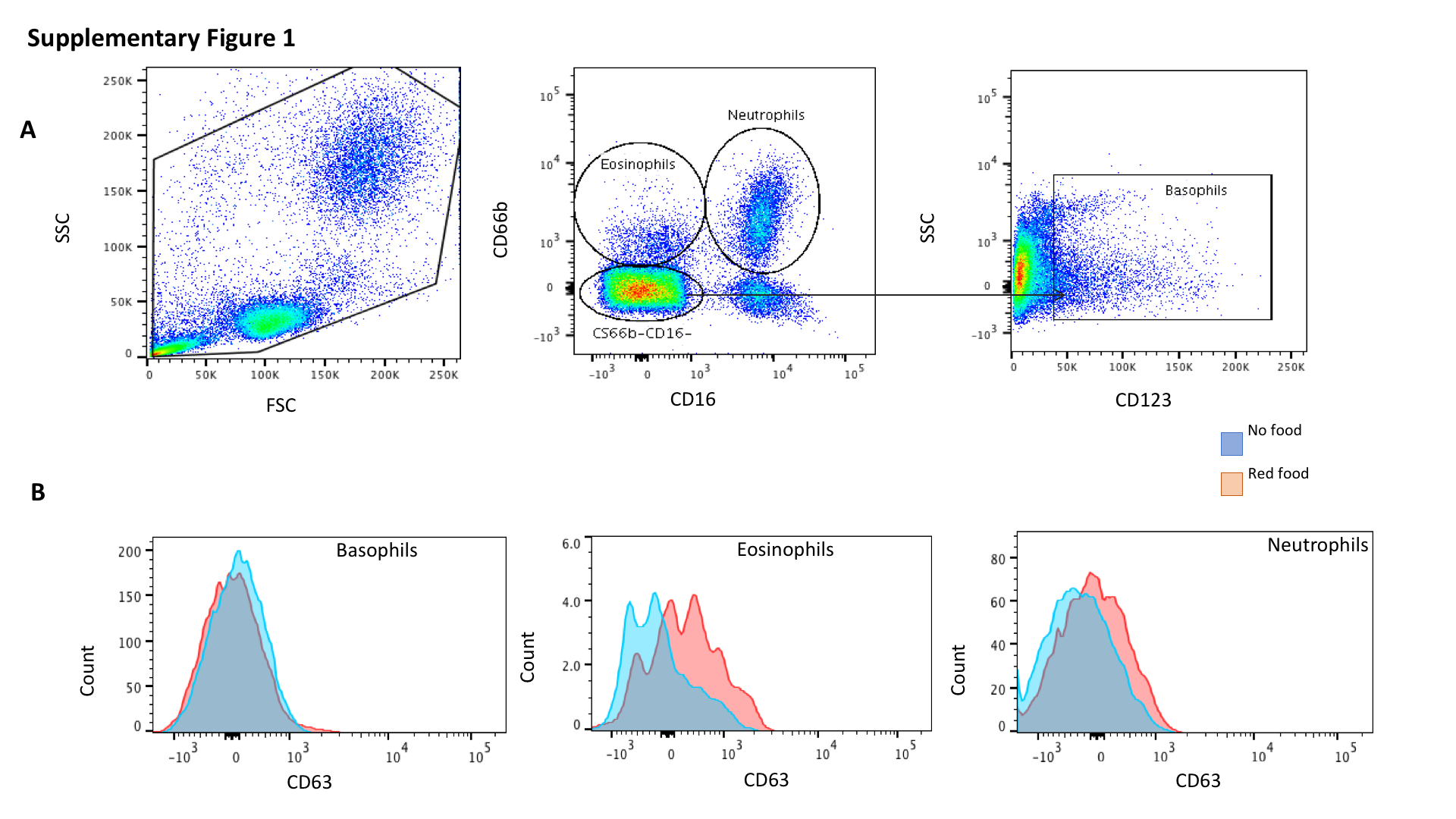

Supplement: Supplementary file 4 — Figure S1. Representative cell gating and CD63 expression. (A) Gating of total cells, eosinophils, neutrophils and basophils for a representative sample. (B) Basophils, eosinophils and neutrophils CD63 expression for a representative sample no treated (no food) or treated with a positive food (red food). (TIFF 8103 kb) [file 12986_2018_260_MOESM4_ESM.tiff]
